# EXPECTATIONS, ROLES, AND EXPERIENCES OF GRANDPARENT-CAREGIVERS OF CHILDREN WITH RARE DISEASE

**DOI:** 10.1093/geroni/igz038.3501

**Published:** 2019-11-08

**Authors:** Jasmine Manalel, Betina Hollstein, Laura M Koehly

**Affiliations:** 1 National Institutes of Health, Bethesda, Maryland, United States; 2 University of Bremen, Bremen, Germany, Germany; 3 National Human Genome Research Institute, National Institutes of Health, Bethesda, Maryland, United States

## Abstract

Grandparenting can be a rewarding and health-promoting experience for older adults. However, grandparent-caregivers often experience greater stress and poorer health relative to non-caregiving grandparents. Further, little is known about grandparents caring for a child with a rare, chronic illness. This study aimed to extend knowledge of the expectations, roles, and experiences of grandparents providing care to a child affected with an inherited metabolic condition. The sample included 23 grandparent-mother dyads from the Inherited Diseases, Caregiving, and Social Networks Study. The grandparent sub-sample ranged from 49 to 79 years of age (Mage = 64), the majority were female (83%) and married (74%), and almost half (48%) were retired. Social network assessments were analyzed to determine concordance between mother- and grandparent-reports of grandparents’ role in the child’s caregiving network. Fifteen mother-grandparent dyads (65%) agreed on grandparents’ role in the child’s network, with the majority of those (93%) considering the grandparent to be very close and important (versus less close or excluded from the caregiving network). Grandparents whose self-reports of their role in their child’s caregiving networks were consistent with mother-reports appeared more likely to report that they spend enough time caregiving than those whose reports were inconsistent. Content analysis of grandparents’ interviews provided supporting information about the joys and regrets of their grandparenting experience and perspectives on caregiving expectations. This research leverages multi-informant social network and qualitative data to illuminate grandparents’ role in the caregiving networks of chronically ill children and adaptation to non-normative grandparenting experiences.

